# Strategies to Engage Adolescents in Digital Health Interventions for Obesity Prevention and Management

**DOI:** 10.3390/healthcare6030070

**Published:** 2018-06-21

**Authors:** Stephanie R. Partridge, Julie Redfern

**Affiliations:** 1 Faculty of Medicine and Health, Westmead Applied Research Centre, The University of Sydney, Westmead, NSW 2145, Australia; julie.redfern@sydney.edu.au; 2 Faculty of Medicine and Health, Sydney School of Public Health, Prevention Research Collaboration, Charles Perkins Centre, The University of Sydney, Camperdown, NSW 2006, Australia; 3 The George Institute for Global Health, The University of New South Wales, Camperdown, NSW 2006, Australia

**Keywords:** engagement, adolescents, obesity, diet, prevention, management

## Abstract

Obesity is one of the greatest health challenges facing today’s adolescents. Dietary interventions are the foundation of obesity prevention and management. As adolescents are digital frontrunners and early adopters of technology, digital health interventions appear the most practical modality for dietary behavior change interventions. Despite the rapid growth in digital health interventions, effective engagement with adolescents remains a pertinent issue. Key strategies for effective engagement include co-designing interventions with adolescents, personalization of interventions, and just-in-time adaptation using data from wearable devices. The aim of this paper is to appraise these strategies, which may be used to improve effective engagement and thereby improve the dietary behaviors of adolescents now and in the future.

## 1. Introduction

The burden of obesity and its related comorbidities is one of the most significant health challenges facing today’s youngest generation [1]. In 2016, 18% of the global population of children and adolescents had overweight or obesity and the prevalence of adolescent (10–19 years) overweight and obesity are increasing [2]. Weight gain during adolescence is associated with cardiovascular disease in later life [3,4]. Adolescents who gain weight and maintain a high body mass index (BMI) into adulthood, have higher odds of developing hypertension and systemic inflammation [3,5,6]. Management of obesity during adolescence is challenging as greater than 90% of adolescents with obesity will transition to adulthood remaining overweight or obese [7,8]. This is a significant concern as there are over 1.8 billion young people between the ages of 10 and 24 years, accounting for the largest generation in history [9]. Innovative, contemporary and engaging dietary interventions are needed to prevent and manage overweight and obesity, particularly in adolescents, whose specific needs are often unrecognized by healthcare providers.

Dietary interventions are the foundation of obesity prevention and management. Adolescents need engaging interventions, as they are not achieving dietary intake recommendations. This is concerning as poor nutritional behaviors are linked to one in five deaths, globally [10]. For example, in Australia in 2015, less than 1% of adolescents eat enough vegetables, less than 27% eat enough fruit, and less than 2% eat adequate amounts of high-calcium foods [11]. They were also the highest consumers of convenience foods, such as discretionary foods and sugar-sweetened beverages [12]. Adolescents face exposure to an overabundance of highly palatable convenience foods, which can result in excessive energy intake [13]. Such excess energy intake is often in combination with a decline in physical activity and an increase in sedentary behaviors during the transition from childhood to adolescence, thereby reducing their total energy expenditure [14]. The result is positive energy balance and subsequent weight gain. Weight gains of 1–5 kg per year, in addition to normal adolescent growth, can result from consuming as little as 84–418 kilojoules (kJ) (20–100 kilocalories (kcal)) per day more than expended [15,16]. Despite the debate about optimal macronutrient composition for weight management, national bodies have agreed achieving neutral or negative energy balance is the most critical factor affecting weight maintenance or loss, respectively [17,18]. It is therefore essential adolescents’ dietary interventions for both obesity prevention and management are engaging and support sustainable long-term improvements in dietary behaviors.

There has been rapid growth in research using digital technologies for behavior change in the areas of physical activity, sedentary time and diet [19]. Digital behavior change interventions, defined as “a product or service that uses computer technology to promote behavior change” [20], use various technologies for delivery such as websites, social media, text messages, smartphones apps or wearable devices [20,21,22]. As adolescents are one of the highest users of technology [23], their online digital environment can be congested. It is, therefore, imperative researchers and clinicians are implementing strategies within the design and delivery of their digital health interventions to engage and capture the attention of adolescents effectively.

Given adolescents are technology frontrunners; digital health interventions appear to be a practical modality for dietary behavior change interventions for the prevention obesity [24,25,26]. We acknowledge digital interventions cannot replace the multifaceted treatment approached required for management of obesity in adolescents [27]. However, digital technologies show potential as an additional tool for weight-loss maintenance following obesity management [28,29,30]. In this paper we review the evidence supporting effective engagement in digital interventions as a critical factor in the adoption of healthy dietary behaviors in adolescents within the current “digital world” [31]. We then narratively review three key strategies that researchers and clinicians can use to promote engagement and thereby potentially increase the effectiveness of digital dietary interventions for the prevention of obesity and maintenance of weight-loss in adolescents. We selected three strategies, namely, co-design, personalization, and just-in-time adaptation, given the feasibility and practicality of these strategies for both researchers and clinicians working in obesity prevention and management.

## 2. Adolescents’ and Their Digital World

Adolescence is the period of transition between childhood and adulthood, characterized by the complex interplay of biological growth, cognitive development and social role transitions [32,33]. Puberty is a key event in early adolescence resulting in rapid changes in body composition and subsequently dietary requirements [34]. The World Health Organisation (WHO) defines an adolescent as a person aged between 10–19 years [1]. Given the variability in onset and duration of puberty and the changing social environment, it has been suggested 10–24 years maybe more representative of the adolescent period [35]. Regardless, adolescence is a critical life stage to intervene for the establishment of healthy dietary behaviors and to ensure overall health and lower mortality risks in later life [7,8].

Inadequate nutrition, during adolescence, may compromise growth and development with long-term consequences, such as overweight and obesity. Adolescents have different nutritional needs according to their age, gender, stage of physical maturity and level of physical activity, however, requirements for all nutrients increases dramatically during puberty [36]. During adolescence, total energy (kilojoule, kJ), protein and some micronutrient requirements are lower than that of adults. However, per kilogram (kg) relative to their total body size, energy, macronutrients and micronutrients requirements are higher than that of adults [36]. Similarly, per kJ relative to their total energy requirements, macronutrients and micronutrients requirements are also higher than that of adults [36]. For example, boys aged 13 years usually require 29 milligrams (mg) of calcium per kg of body weight, compared to adult males, who need only 14 mg of calcium per kg of body weight [37]. It essential during this time of growth adolescents are consuming a nutrient dense diet and are forming healthy dietary behaviors and developing weight regulation strategies to carry forward into adulthood.

Engaging adolescents in obesity prevention or management programs to improve their dietary behaviors remains a crucial challenge. Adolescence is often a busy life stage. Along with school, adolescents’ schedules can include additional activities such as study, extracurricular activities, part-time work and social events, all of which can complicate recruitment and engagement efforts. Current attrition rates for obesity management in children and adolescents are highly variable, suggesting between 27% and 73% of participants drop out of interventions [38]. There is emerging evidence suggesting researchers and clinicians need to initially engage adolescents by using positively framed messaging [39,40] with preferred weight terminology [41], as the stigma associated with being overweight or obese is a significant barrier for adolescents to seek out health services. Also, it is important to prioritize accessibility and enjoyment in the design phase of dietary interventions [39]. Digital technologies for obesity prevention and management can play a key role in addressing accessibly and enjoyment for adolescents, as well as to widely distribute positive messages to recruit adolescents.

The ubiquitous infiltration of technology in the lives of adolescents offers a potential opportunity for capitalizing on digital technology as a feasible and acceptable modality for dietary interventions to prevent and manage obesity. The current generation of adolescents (‘Generation Z’), i.e., those born after 1995, are creating the most global youth culture in history and most have access to similar digital technologies. In Australia, over 90% of adolescents aged 14–17 years own a mobile phone, and 94% of those own a smartphone [42]. Adolescent smartphone ownership in Australia is higher than that of their counterparts in the United States (73%) and United Kingdom (69%) [42]. In developed countries, 83% of adolescents go online three or more times per day, text messaging is their primary form of mobile phone communication and they are one of the highest users of social media and smartphone applications (‘apps’) [23]. Digital health interventions for overweight and obesity in adolescents can result in improvements in BMI and lifestyle outcomes, including dietary behaviors, in the short-term (less than 6-months) [43,44,45]. Thus, adolescents are immersed in a ‘digital world’ and given the emerging short-term evidence this is likely to offer a further opportunity for incorporating dietary interventions into digital technologies.

## 3. Three Strategies for Effective Engagement with Digital Intervention

**Figure 1:**
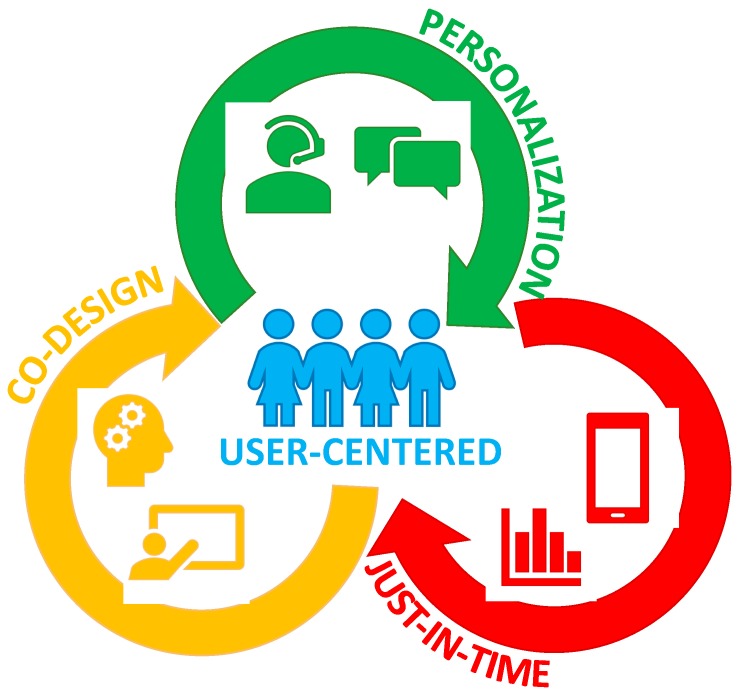
A conceptual illustration of the interaction between the three user-centered strategies, namely, co-design, personalization, and just-in-time adaptation.

Effective engagement with digital health interventions is essential for effective behavior change. The complexity of engagement with digital interventions, which target various health-related behaviors has led to different conceptual models. A recent systematic review by Perski et al. [46], synthesized the literature on engagement and developed a conceptual framework of direct and indirect influences on engagement with digital health interventions. Moreover, in a recent publication by Yardley et al. [47], the authors presented a figure to conceptualize the closely linked and mediating relationship between engagement with digital technology and behavior change, at both micro and macro levels. In addition, Yardley and colleagues present a range of available methods to measure effective engagement [47]. Despite the current challenges about how to best define and measure engagement with digital health interventions [46,47], experts agree that effective intervention design requires a user-centered and iterative approach [47,48]. As well, researchers have identified behavior change techniques embedded within adolescent obesity prevention and management interventions which may contribute to effectiveness [40]. Considering this, we will now examine three strategies to increase effective engagement with digital health interventions to improve dietary behaviors. These interacting, user-centered strategies are co-design, personalization, and just-in-time adaptation. We present a conceptual illustration of these three strategies in Figure 1.

### 3.1. Co-Design

Co-design or participatory design in public health is defined as the systematic co-creation, with those affected by the issues being studied, for the purpose of developing new strategies, programs, policies [49,50]. Co-design is an umbrella term used to describe the array of approaches that can be utilized to engage the end-users (i.e., those affected by the issue being studied) or other stakeholders in the research process [49]. Ideally co-design can be thought of as the ‘golden thread’ that runs through all stages of research, from design to implementation in real-world settings. It is the collective sum or a framework of these approaches which constitutes co-design, not the use of individual methods in isolation, such as focus groups or interviews [51]. However, given the rapid pace of digital technology development, and short research funding cycles, researchers and clinicians are using commercial apps for adolescent weight management that do not include evidence-based strategies and have not been co-designed with adolescents [52,53]. Considering adolescents are digital frontrunners, their lack of input into technologies to manage their own health and wellbeing is likely to result in ineffective levels of engagement.

Available frameworks [51] and findings from co-design research in adolescent mental health and primary care can guide the development of digital health interventions to address effective engagement with adolescent obesity prevention and management interventions. Two recent Australian research studies have described a co-design process to develop apps to improve young people’s experience of seeing their general practitioner [54] and for self-monitoring and management mood symptoms in adolescents with depression [55]. A similarity of both studies throughout the co-design process was the identification of contrasting needs, motivations and intentions for the apps between researchers, clinicians, and adolescents [54,55]. However, the co-design method facilitated a process of mutual learning of each group’s needs and expectations, with the emphasis on designing from the perspectives of the adolescent (‘end user’).

Two recent studies utilize co-design approaches for the development of digital technologies to address adolescent overweight and obesity [56,57]. Through a co-design process to develop a smartphone app to support weight and health management, Rivera et al. [56] were able to identify adolescents require personalized assistance with meal planning, including more convenient and efficient ways to plan meals and make healthier food choices throughout the day. This feature is not available in current commercial apps, which predominately focus on self-monitoring and caloric monitoring of food intake [58]. Moreover, Standoli et al. [57] found adolescents were interested in monitoring their daily activities by using wearable devices or clothing. However, the short lifespan of currently available commercial activity trackers was a significant barrier. The researchers and adolescents were able to co-design smart clothing items to monitor daily activities that were acceptable, personalized and met the needs of adolescents. Thus, these examples, albeit limited to smartphone apps, show co-design increases the likelihood of acceptable digital health technologies and subsequently may result in effective engagement in future interventions in both research and real-world settings.

### 3.2. Personalization

Personalization or tailoring is a common theme that emerges in the co-design process and also is a key component of effective dietary interventions [59,60]. Personalization in dietary interventions and healthcare in general, goes beyond recommending population-based guidelines to using such guidelines to develop individualized management plans [61]. As alluded to in our introduction, personalization is a key feature of the multifaceted face-to-face treatment approach required for management of obesity in adolescents [27,62]. At the present time, such personalization for obesity management is unlikely to be replicated fully in digital interventions. However, semi-personalization is presently achievable within digital interventions for obesity prevention and weight-loss maintenance following obesity management [63]. Digital interventions, such as text messaging programs, can provide semi-personalized messages to positively change individual lifestyle behaviors [64]. Large populations of people can also be targeted simultaneously, as text messages are a low-cost, convenient, and scalable method of health communication.

As text messaging remains a primary form of communication between adolescents, semi-personalized text messages, constructed carefully in collaboration with adolescents, have been shown to be a feasible and acceptable form of communication for obesity interventions [63,65,66]. High-quality evidence for the effect of text messages on BMI in both overweight and obesity adolescent populations is lacking [24,26]. The findings from two randomized controlled trials provide insights about the role of semi-personalized text messages in changing dietary behaviors and subsequently reducing in BMI. The multicomponent mobile health study in young adults by Allman-Farinelli et al. [67,68], used eight weekly motivational text messages based on the Transtheoretical Model of Behavior Change, whereby messages matched the stage-of-change for each lifestyle behavior at baseline. Text messages were delivered in conjunction with health coaching calls, a website and smartphone apps. Young adults in the intervention group weighed 3.7 kg (95% confidence interval (CI) −6.1, −1.3) less at 3-months, and 4.7 kg (95% CI −6.9, −1.8) less at 9 months [67] compared to their control counterparts. Further, intervention participants consumed more vegetables (*p* = 0.009), fewer sugary soft drinks (*p* = 0.002), and fewer energy-dense takeout meals (*p* = 0.001) compared to controls [68]. The process evaluation from the study found intervention participants valued the text messages and found the text messages increased their overall engagement with the program [69]. The study by Chow et al. used a multistep, iterative, mixed methods process with heart disease patients to develop text messages that provide semi-personalized information, motivation, and support to meet national guidelines for heart disease. Intervention participants significantly reduced their BMI at 6-months (−1.3 kg m^−2^ (95% CI −1.6, −0.9, *p* < 0.001) [22]. Moreover, a significantly higher proportion of intervention participants adhered to greater than four dietary guideline recommendations compared to the control group (93% vs. 75%, *p* < 0.001) [70]. Patients reported the semi-personalized text messages increased engagement and supported their behavior change [21]. Further research is required to see if the two semi-personalized text messages examples presented here can be applied to prevention of obesity or for weight-loss maintenance following obesity management in adolescent populations.

### 3.3. Just-in-Time-Adaptation

Just-in-time adaptive interventions are a form of personalized interventions that provide support relevant to an individual’s changing behaviors and contexts over time [71]. The overall goal is to provide instantaneous contextual support for the targeted behaviors when the individual is most likely to be receptive. Just-in-time adaptive interventions use sensory data, e.g., a smartphone or smartwatch and momentary information directly from participants, e.g., ecological momentary assessments (EMAs), to send personalized feedback on targeted behaviors [72]. In these interventions, text messages commonly communicate the behavioral feedback. A recent systematic literature review of just-in-time-interventions found behavioral feedback that was always available, personalized, and practical resulted in significant positive behavioral changes [73].

Only a few studies have been conducted, which describing the potential role of interactive digital health interventions for adolescents [72,74]. One example is the KNOWME study, by Spruijt-Metz and colleagues, which demonstrated the feasibility and acceptability of a just-in-time adaptive intervention for overweight adolescents [72,75]. KNOWME study aimed to reduce sedentary behavior and promote physical activity. The pilot study showed adolescents decreased their sedentary time by 170.8 min per week compared to baseline (*p* < 0.01) and physical activity levels measured via accelerometers were found to be significantly higher after receiving text messages with feedback from the research team (*p* < 0.01) [72]. Pilot research by Garcia et al. [76] developed a feasible youth EMAs via a two-way text message system to collect information on daily activities, behaviors, and attitudes among adolescents. Adolescents live in an instantaneous and fast-paced digital environment. Therefore, such interventions show significant potential.

## 4. Conclusions

Engagement with digital health interventions is an important mediating factor to improve dietary behaviors and prevent and manage obesity in adolescents. The rapid development and diffusion of digital health interventions for adolescents has resulted in few interventions that are co-designed with end-users, personalized and provide real-time feedback. Incorporating such strategies may optimize the levels of engagement adolescents have with digital health interventions to improve their dietary behaviors. Strategies to increase engagement are not limited to those discussed in this narrative review. There are several other strategies that have the potential to increase engagement with digital interventions in other populations with different needs. Given the emerging body of evidence suggesting effective engagement with digital health interventions can mediate positive behavioral change, research efforts should be focused on incorporating engagement strategies throughout the research process and as well in real-world scaled up digital health interventions and programs.
